# Creating a 3D microenvironment for monocyte cultivation: ECM-mimicking hydrogels based on gelatine and hyaluronic acid derivatives†

**DOI:** 10.1039/c7ra13739g

**Published:** 2018-02-16

**Authors:** Julie Bystroňová, Ivana Ščigalková, Lucie Wolfová, Martin Pravda, Nihal Engin Vrana, Vladimir Velebný

**Affiliations:** Contipro a.s. Dolni Dobrouc 401 56102 Dolni Dobrouc Czech Republic martin.pravda@contipro.com; Protip Medical 8 Place de l'Hôpital 67000 Strasbourg France; Inserm UMR 1121 11 rue Humann 67085 Strasbourg France

## Abstract

Macrophages play a critical role in the initial response to foreign materials in the body. As most biomaterial-based implantable devices would be treated as a foreign body by the immune system, there is a need for systems that can establish a favourable interaction between the implanted biomaterial and the host. Herein, we describe such a system that can be used as an ECM-like microenvironment for macrophage polarization. The hydrogel system was designed to provide a co-crosslinkable microenvironment containing both protein and glycosaminoglycan components, a hydroxyphenyl derivative of gelatine (GTN–HPA) and tyraminated hyaluronic acid (HA–TA). Both polymers can undergo a crosslinking reaction between polymer chains *via* the same polymerisation initiation system where the polymer network is formed by crosslinks between phenols in GTN–HPA and HA–TA. The mechanical properties and swelling of the hydrogel can be easily controlled as a function of the crosslinking mode and by the ratio of GTN–HPA and HA–TA compounds used. THP-1 monocytes were successfully encapsulated in the gels and cultured for up to 28 days. Cells exhibited higher metabolic activity when encapsulated in softer hydrogels (*E* ≈ 10 kPa) compared to stiffer (*E* ≈ 20 kPa) material in which monocytes tended to form large clusters. Encapsulation of monocytes in the material with HA–TA content enhanced the expression of macrophage-related genes. We demonstrated a co-crosslinkable GTN–HPA and HA–TA matrix microenvironment that is suitable for *in vitro* micro tissue model applications.

## Abstract 2

A co-crosslinked gelatin and hyaluronan based matrix is a suitable microenvironment for the 3D culture of THP-1 cells, which enhances their differentiation into macrophages.

## Graphical abstract

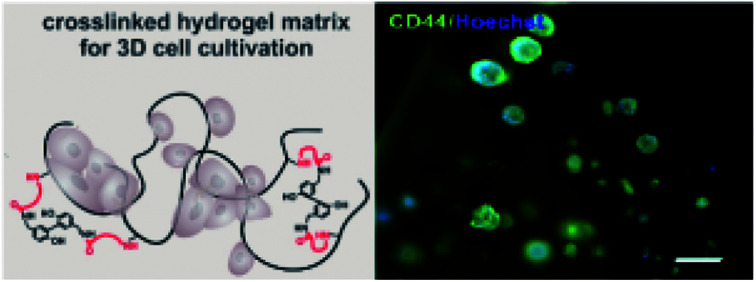

## 1. Introduction

Implants, transplants, active devices and tissue-engineered structures are current solutions to many debilitating diseases and conditions that would otherwise result in organ damage or dysfunction. However, the innate and adaptive immune systems of the human body pose a significant problem for their application, as the immune system has evolved to defend the body against all foreign bodies and it cannot distinguish between a therapeutic device, detrimental foreign body or infection. The inflammatory response that in many cases follows implantation can lead to chronic inflammation or, in extreme cases, rejection and destruction of the implanted system.^1^ In the case of transplants or allogenic biomaterials, this problem is generally overcome with immunosuppressants which, however, have the adverse side effect of weakening the immune system against other dangers such as cancer and infection. For biomaterials in general, one solution is to develop immunomodulatory coatings that will direct and control the immune response upon implantation. These coatings can be anti-inflammatory^2^ by virtue of their own activity or by acting as a controlled-release system for anti-inflammatory molecules.^3^

One other possibility is to use patient's own immune cells to control the immune response to the implanted material. This is especially pertinent for cell-based therapies such as tissue engineering.^4,5^ Macrophage phenotype plasticity is an important part of their ability to respond to different kinds of injuries. During the healing process, there are two types of macrophages present in the body following the initial injury: the M1 type macrophage (M1) is present in the early stages of wound healing and induces the inflammatory process. Later, M1 switches to the M2 type macrophage (M2) that induces the tissue healing process and supports cell proliferation.^6–8^ The switch from M1 to M2 at the resolution phase of inflammation induces the establishment of a microenvironment suitable for tissue healing and repair. Artificial induction of such environment around implanted materials using autologous immune cells can facilitate the integration and remodelling of the implanted material.^9^

As the use of biomaterial/cell combined systems in 3D has become more and more common, the emphasis on the mimicking of the 3D microenvironment of the extracellular matrix has increased.^10^ One of the main ways of ECM-mimic structures design is the use of the derivatives of the actual ECM components. Hyaluronan (HA) is a natural glycosaminoglycan, one of the chief components of the extracellular matrix, and it is involved in the regulation of wound healing, angiogenesis and immunomodulation.^11–15^ Gelatine (GTN) is also a natural polymer, prepared by a partial hydrolysis of collagen, the chief component of connective tissues. Both polymers exhibit highly hydrophilic character and are commonly used for preparation of hydrogels.^16^ Hyaluronic acid–tyramine conjugate (HA–TA) and GTN modified by hydroxyphenylpropionic acid (HPA) were recently described as biopolymer derivatives for preparation of covalently crosslinked hydrogels suitable for application in the field of tissue engineering and regenerative medicine.^17,18^ Thanks to presence of hydroxyphenyl moieties, both modified biopolymers undergo horseradish peroxidase mediated co-crosslink which can be performed under physiological condition in the presence of living cells.^19^

The present study deals with the development of a hydrogel material that would serve as a scaffold for the incorporation of monocytes. The material should provide suitable 3D matrix for their successful encapsulation, cultivation, and proliferation. Moreover, developed hydrogel should enable a control of the differentiation of entrapped monocytes. Such system could be used as suitable implant coating for control of inflammatory response of recipient tissue.^20^

## 2. Materials and methods

Hyaluronan polyaldehyde was provided by Contipro a.s. 3-(4-hydroxyphenyl)propionic acid (HPA), 2-(*N*-morpholino)ethanesulfonic acid hydrate (MES), *N*-(3-dimethylaminopropyl)-*N*′-ethylcarbodiimide hydrochloride (EDC), *N*-hydroxysuccinimide (NHS), gelatine from porcine skin gel (Bloom strength of 300; Type A), dimethylformamide, tetrahydrofuran, ethylacetate, methanol, trifluroacetic acid, and horseradish peroxidase – type I (HRP) were purchased from Sigma Aldrich. Hydrogen peroxide 30% was purchased from Penta s.r.o.

### 2.1. NMR analysis

Solution-state NMR spectroscopy was carried out on a Bruker Avance III 500 MHz instrument operating at a proton frequency of 500.25 MHz and a carbon frequency of 125.80 MHz. The spectrometer was equipped with a 5 mm Bruker BBFO plus broadband probe with an actively shielded *z*-gradient coil. All spectra were acquired and elaborated by the Bruker 2.1 Topspin software. Freeze-dried samples (7 mg) were dissolved in D_2_O (0.75 ml) and transferred into 5 mm NMR quartz tubes.

### 2.2. Molecular weight determination

The molecular weight of the HA–TA conjugate was assessed using SEC-MALLS (size exclusion chromatography/multiangle laser light scattering). Samples were dissolved overnight in a mobile phase to produce solutions with a concentration of 2 mg cm^−3^. The chromatographic system consisted of an LC-10ADVP Shimadzu HPLC pump, SIL-10AF autosampler, CTO-10AVP column oven, SCL-10AVP system controller, DGU-14A degasser, RID-10A refractive index detector, SPD-10AVVP UV-VIS detector (all from Shimadzu), and a miniDAWN TREOS light scattering photometer (Wyatt Technology Corporation). The injection volume was 100 μl. Each sample was filtered through a 0.22 μm MS Nylon Syringe Filter. The mobile phase consisted of aqueous 50 mM sodium phosphate and 0.02% sodium azide at a flow rate of 0.8 ml min^−1^. Data acquisition and molecular weight calculations were performed using the ASTRA software (version 5.3.4, Wyatt Technology Corporation, USA). A specific refractive index increment of 0.155 ml g^−1^ was used for HA.

### 2.3. UV/vis spectroscopy

To determine the HPA conjugation degree, UV/vis spectroscopy was performed on a Shimadzu UV-2401PC spectrometer in the range of 250–300 nm. UV spectra were processed by the UV Probe software version 2.00. The total amount of HPA moiety in the modified gelatine was determined by photometric measurement at 277 nm.

### 2.4. Synthesis of hydroxyphenyl-modified gelatine (GTN–HPA)

GTN–HPA was synthesized using a method previously described by Wang *et al.*^21^ 3-(4-Hydroxyphenyl)propionic acid (3.4 g, 20.5 mmol) was dissolved in 50 ml of DMF. MES (10 g), NHS (3.5 g, 30 mmol) and EDC (3.9 g, 20.5 mmol) were dissolved in 100 ml of demineralized water. Both solutions were mixed together and stirred for 60 minutes at room temperature. Simultaneously, GTN (10 g) was dissolved in demineralized water (500 ml) by heating it to 40 °C. All components were finally mixed and stirred overnight at room temperature. The GTN–HPA conjugate was purified by ultrafiltration and isolated by freeze-drying. Modification of GTN was confirmed by ^1^H NMR and DOSY spectroscopies. The content of HPA in the conjugate structure was determined by UV/vis spectrophotometry.

### 2.5. Preparation of hyaluronan–tyramine conjugates (HA–TA)

#### Synthesis of 6-amino-*N*-[2-(4-hydroxyphenyl)ethyl]hexanamide (Ahx–TA)

6-[(*tert*-Butoxycarbonyl) amino] hexanoic acid (1.00 g, 4.3 mmol) was dissolved in 50 ml of tetrahydrofuran (THF). A solution of 1,1′-carbodiimidazol (0.70 g, 4.3 mmol) was then added. The mixture was heated to 50 °C for 1 hour. The reaction vessel was subsequently washed with an inert gas. Tyramine (0.59 g, 4.3 mmol) was added to the reaction mixture. The mixture was then heated for another 2 hours. THF was subsequently removed by means of reduced-pressure distillation. The evaporation residue was dissolved in 50 ml of ethyl acetate. The solution was washed with 150 ml of purified water (divided into three parts). Ethyl acetate was removed by means of reduced-pressure distillation. The evaporation residue was dissolved in 50 ml of MeOH, and 2 ml of trifluoroacetic acid (TFA) were added. The solvent was removed by means of reduced-pressure distillation. The product was isolated as colourless oil. The structure of the product was confirmed by ^1^H NMR spectroscopy.

#### HA–TA synthesis

Hyaluronan polyaldehyde^22^ (5.00 g, 12.50 mmol dimers of HA, 450 kDa, DS = 9%) was dissolved in 500 ml of demineralized water. The pH of the solution was adjusted to 5 using acetic acid. 6-Amino-*N*-[2-(4-hydroxyphenyl)ethyl]hexanamide (1.25 mmol) was added to the reaction mixture and the mixture was then stirred for 1 hour at room temperature. Afterwards, a solution of picoline–borane complex (0.27 g, 2.5 mmol) in 50 ml of 50% propan-2-ol was added to the mixture. The reaction mixture was subsequently stirred for 12 hours at room temperature. Low-molecular ballast substances were removed from the product by means of ultrafiltration. The product was obtained after precipitation by propan-2-ol.

The structures of the HA–TA conjugates were confirmed by ^1^H NMR spectra. ^1^H NMR was also used to determine the degrees of substitution (DS) of the prepared hyaluronan–tyramine conjugates. The DS was calculated according to equation:
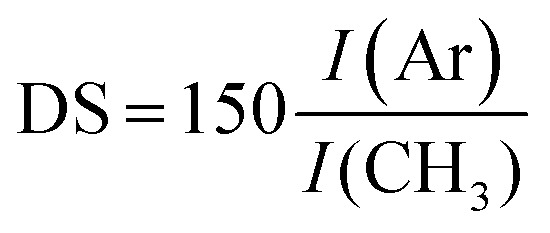
where *I*(CH_3_) is an integral of the signal at 2.01 ppm (methyl of the *N*-acetyl group) and *I*(Ar) is an integral of signals of two aromatic protons at 6.49 ppm (for HA–TA).

### 2.6. Evaluation of hydrogel properties

Hydrogel samples were prepared from 4% (w/v) solutions. Different ratios (w/w) of compounds were used (always presented in this order: HA–TA : GTN–HPA). HA–TA with *M*_w_ 450 kDa and DS 2% was used in combination with GTN–HPA with HPA substitution (15 mg g^−1^). For sample preparation, HA–TA derivative was dissolved while being constantly stirred at room temperature. Then the gelatine in the specified amount was added to the solution and dissolution was performed in a heated water bath at 37 °C over 2 hours.

The two-compound system was used for hydrogel preparation. The solution was divided into two parts of equal volume into which the reaction-mediating agents were dosed separately. The HRP stock solution had an activity of 8 U ml^−1^. The H_2_O_2_ solution was dosed at a concentration of 0.1%. Afterwards, equipment with a static mixer was used for a homogeneous mixing of both parts. It allowed to perfectly homogenize both solutions and dose the material into Teflon moulds. Gels formed within seconds; samples were allowed to set in the moulds for 15 minutes at room temperature. Round-shaped samples were prepared with diameter 11 mm and app. volume 0.4 ml.

The stiffness of the hydrogels was evaluated by measuring the Young's modulus in compression on an Instron 3343 (load cell 100 N, crosshead speed 2 mm min^−1^; 0.01 N pre-compression of the sample achieved at a crosshead speed 500 μm min^−1^). The Young's modulus was calculated from the slope of the first 10% of strain. Collected data were evaluated by Bluehill®3. Each measurement was performed in five replicates.

The swelling ratio of the samples was determined from their weight increases in the swollen state; these tests were performed under sterile conditions. The tested hydrogel samples were weighed, immersed in 4 ml of saline and stored at 37 °C. After four days, samples were gently blotted dry and weighed in their swollen state. The swelling ratio (w/w) was calculated from the ratio of weight after and before swelling,^23^ and, additionally, mechanical properties of the swollen samples were evaluated.

The gelation time was measured on a TA Instruments AR-G2 rheometer. The settings for the rheology tests were as follows: parallel plate (40 mm), gap 400 μm, dosed volume 525 μl, temperature 37 °C, mode “time sweep step” (frequency 1 Hz and displacement 0.001 rad). 500 μl of the derivative solution with HRP (0.24, 0.4, and 0.56 U ml^−1^) were dosed on the lower plate, and 25 μl of the H_2_O_2_ solution in adequate concentrations (19.8, 33, and 46.2 mM) were dosed separately; pre-shear 2000 1/s for 1 s was used for the homogenization of the compounds. The gelation times (*T*_g_) were determined from the intersections of the *G*′ and *G*′′ moduli; the analyses were carried out on the Trios software.

### 2.7. THP-1 encapsulation and cultivation within the hydrogel

A THP-1 monocytic cell line (ATCC® TIB-202™) was used for the purposes of this work. Culture medium (CM) contained a high-glucose RPMI medium supplemented with 10% FBS, 1% penicillin–streptomycin solution, 2 mM l-glutamine, and 1 mM sodium pyruvate (all from Sigma Aldrich).

1.5 × 10^6^ THP-1 cells were encapsulated per ml of hydrogel with respect to the concentrations of crosslinking agents specified in Section 2.6. The solutions were sterilized by filtration. Cells were added to the solution aliquot with HRP. The solution was gently stirred to homogenize cells, and hydrogel samples were prepared and left to set for 15 minutes. Finally, hydrogels with encapsulated cells were immersed in the culture medium and cultured for a set amount of time. Non-encapsulated cells were used as control.

#### Evaluation of monocyte- and macrophage-mediated hydrogel degradation

THP-1 cells were encapsulated in hydrogels and cultured for 14 days. A standard CM was used for the first 24 h (day 0–day 1). Cells were differentiated (protocol adapted from^24,25^) into macrophages by treating them with 100 nM phorbol 12-myristate 13-acetate (PMA, Sigma Aldrich) over the next 24 h (day 1–day 2) followed by a 24 h incubation in CM (day 2–day 3). Finally, cells were differentiated with 20 ng ml^−1^ IL-4 (M2 polarization) over 72 h (day 3–day 6). The rest of the cultivation was performed in CM (day 6–day 13). Control cells (non-encapsulated and encapsulated) were cultured in CM for the whole 13 days. Hydrogels without encapsulated cells were prepared and treated in the same way as the cell-laden material and served as a control.

### 2.8. Assessment of cell growth and differentiation

#### Live/dead staining

Hydrogels with encapsulated cells were transferred to a 48-well culture plate with 500 μl of PBS. Calcein-AM and propidium iodide staining solutions (both from Sigma Aldrich) were added, both at a final concentration of 1 ng ml^−1^. All samples were incubated for 30 min, protected from light. Photographs were taken on a Nikon Eclipse-Ti fluorescent microscope.

#### Metabolic activity and proliferation

Hydrogels were transferred to a 48-well culture plate; 500 μl of PBS and 100 μl of CellTiter-Glo reagent (Promega) were added. All samples were incubated for 30 min on an orbital shaker at 200 rpm, protected from light. Afterwards, the plate was removed from the shaker and incubated for another 10 min. The luminescent signal (RLU) was read on a TECAN Infinite 200 fluorescent reader. The luminescence value of each sample was normalized to the mass of the hydrogel.

#### Cell cluster size

The diameters of 50 cell clusters were measured for each sample with the NIS-Elements software.

#### RNA extraction, cDNA synthesis and real-time PCR

Scaffolds were harvested at desired time points and frozen at −79 °C for the purpose of gene-expression analysis. After the experiment was finished, RNA was isolated from all the frozen samples: hydrogels were ground up in liquid nitrogen using a pre-chilled mortar and pestle. The obtained powder was transferred to an Eppendorf tube with 600 μl of a pre-warmed CTAB extraction buffer. Further steps were performed according to the CTAB method protocol.^26^

The isolated RNA was reverse-transcribed to cDNA using a High Capacity RNA-to-cDNA Kit. Real time PCR was performed and monitored using a TaqMan® Fast Advanced Master Mix and a TaqMan® Gene Expression Assay (both by Applied Biosystems) according to the manufacturer's instructions. The expression of the HA-receptor CD44 and following genes was assessed: IL-1β, STAT1 (M1 markers) and IL-1RN, STAT6 (M2 markers). The expression level of each target gene was normalized to the expression of the housekeeping gene GAPDH according to the 2^−Δ*C*_t_^ method.^27^

#### Immunofluorescent staining of CD44

Scaffolds harvested on the 28th day were washed in PBS and fixed in 4% paraformaldehyde. Fixed scaffolds were sliced, permeabilized with 0.1% Triton X100 for 5 min and washed several times in PBS. The sections were incubated with mouse anti-CD44 antibody conjugated with FITC (Biotech, 1F-221-T100) in 0.1% BSA for 2 hours. Cell nuclei were counterstained with Hoechst.

### 2.9. Statistical analysis

Statistical analysis was performed using the QC.Expert™ software. Two samples were compared by performing a student's *t*-test. All the data are presented as the mean ± standard deviation. Statistically significant values are presented as *p < 0.05.

## 3. Results

### 3.1. Modification of biopolymers

The hydroxyphenyl moiety was introduced into the gelatine structure according to the standard EDC protocol as it was described by Wang *et al.*^21^ The reaction proceeds as acylation of primary amino groups of lysine residues presented in the gelatine structure by HPA. Modification of the gelatine structure was confirmed by ^1^H NMR and 2D DOSY spectroscopies. The presence of an HPA moiety is indicated by the presence of signals of aromatic protons with shifts 6.5 and 6.9 ppm (see ESI Fig. 1†). 2D DOSY describes differences in the rates of diffusion of molecules in the solution, which is expressed as a diffusion coefficient (see ESI Fig. 2 – *y* axis†). Because of the marked difference in the rates of diffusion of the large molecule of GTN and the small molecule of HPA, the 2D DOSY spectrum can easily indicate whether the low-molecular compound was successfully conjugated with the polymer backbone. The DOSY experiment revealed similar diffusion behaviour of all signals between the GTN backbone (0.88–4.56 ppm) and HPA aromatic protons (6.50 and 6.90 ppm), which indicated that all the proton signals in this region belong to one structural complex where the HPA moiety is covalently bonded to the polymer chain of GTN. The total amount of HPA was determined by photometric measurement (see ESI Fig. 3b†). The prepared GTN–HPA conjugate contained 15 mg of HPA per 1 g of polymer.

The HA–TA derivative was prepared by conjugating modified tyramine (Ahx–TA) with hyaluronan polyaldehyde. A previously published description of the process of reductive alkylation was used for this purpose.^22,28^ The reaction produces a hydrolytically stable secondary amine linkage. Hyaluronan polyaldehyde with a molecular weight of 450 kDa and a degree of substitution of 9% was used. ^1^H NMR spectroscopy was used to confirm the structure of the new synthesized compounds (see ESI Fig. 4 and 5†) and to determine the degree of substitution of the new HA–TA conjugate (DS = 2%, *M*_w_ = 450 kDa).

### 3.2. Optimization of the system for cell cultivation

Preliminary *in vitro* tests showed that the key factor in successful THP-1 encapsulation is the use of a CM in the material precursor solution during the crosslinking of hydrogels (CM – composition is described in Section 2.7). On the other hand, the presence of CM influences the final mechanical properties of the hydrogel. To map this influence, materials were prepared under identical conditions, with the same doses of the crosslinking agents (0.56 U ml^−1^ of HRP and 2.31 mM of H_2_O_2_) and ratio of the polymers 1 : 5 (HA–TA : GTN–HPA), but different solvents were used. Hydrogels prepared from saline had a significantly higher stiffness than materials prepared solely from CM (27.7 compared to 13.2 kPa). Despite their different Young's moduli, the materials swelled almost identically due to syneresis. The weight decreased by app. 10% which corresponds to a similar change in the Young's moduli of the samples after swelling.

**Fig. 1:**
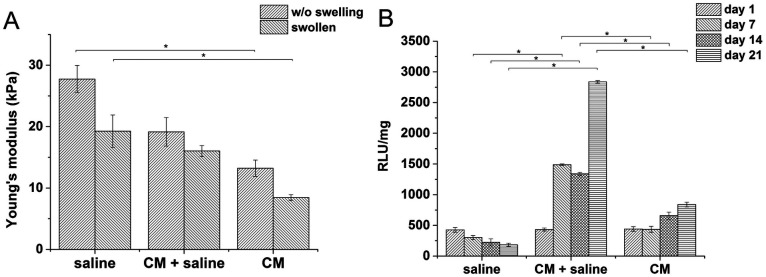
(A) Influence of the type of solvent used on the stiffness of the materials; tested with the same doses of the crosslinking agents; (B) influence of the type of solvent used on the metabolic activity of encapsulated monocytes (*E* ≈ 10 kPa).

The influence of CM content on the viability of encapsulated cells was tested on hydrogel materials of similar stiffness (*E* ≈ 10 kPa) to eliminate the influence of the material itself. There was a significant difference between the viability of THP-1 cells in materials prepared in pure saline, containing pure culture medium, and in a mixture of saline and the culture medium (1 : 1 v/v) (Fig. 1).

### 3.3. Influence of the stiffness of the material on encapsulated cells

As the first step, the concentration of the crosslinking agents was optimized to obtain materials with well-defined stiffness and volume stability. The optimization was performed on materials composed of HA–TA : GTN–HPA at a ratio of 1 : 5.

**Fig. 2:**
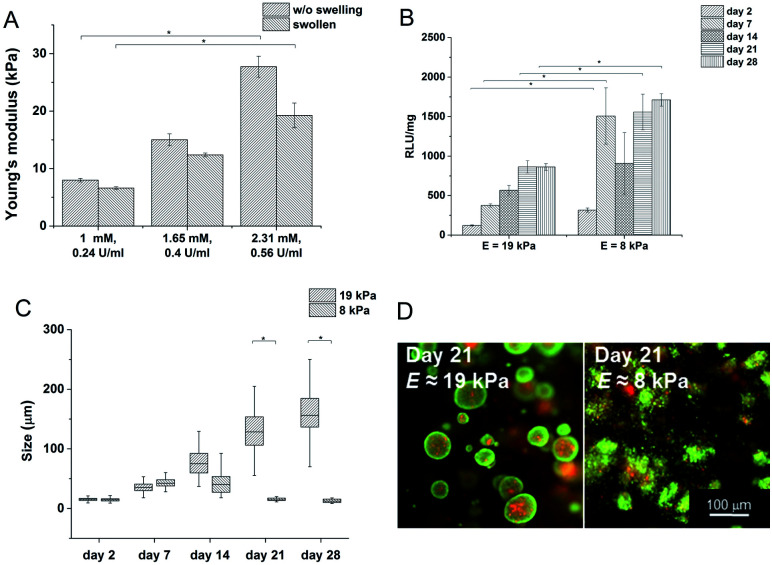
(A) Influence of the concentration of the crosslinking agents on the stiffness of the hydrogels; dependence of the stiffness on (B) metabolic activity; (C) average size of clusters, and (D) cell morphology.

Hydrogels with the lowest stiffness (7.98 kPa) were prepared with 0.24 U ml^−1^ of HRP and 1 mM of H_2_O_2_; the material with the highest stiffness (19 kPa) was prepared with 0.56 U ml^−1^ of HRP and 2.31 mM of H_2_O_2_. The decrease in the Young's modulus after swelling was most substantial at the highest stiffness. But the significant difference between the minimum and maximum values remained even after swelling (Fig. 2A). The gelation time for the lowest concentration of the agents was 9.5 ± 0.15 s; materials with higher concentrations of the agents had gelation time less than 6 s; all materials reached the *G*′ plateau in 3 minutes at all concentrations used.

Materials with the lowest and the highest values of the Young's modulus were chosen for cell encapsulation. Monocytes encapsulated in the stiffer material created clusters with an increased density of cells and had a lower metabolic activity (Fig. 2C) than monocytes in the material with a lower stiffness where the cells created small clusters only during the first 14 days. Later on, cell clusters in the less stiff hydrogels disintegrated into separately growing cells (Fig. 2B and D).

**Fig. 3:**
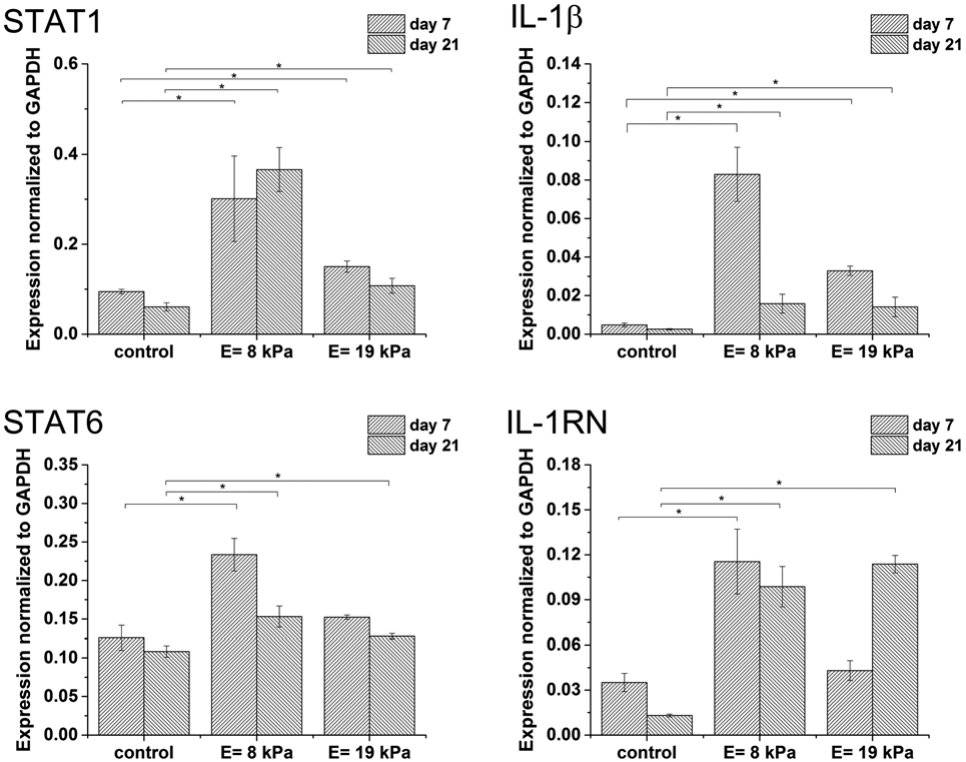
Expression of M1 marker genes STAT1, IL-1β and M2 marker genes STAT6, IL-1RN in non-encapsulated (control) cells and in cells encapsulated in HA–TA : GTN–HPA hydrogels with different degrees of stiffness.

Gene expression was higher in both hydrogels than in non-encapsulated cells (control), suggesting that the incorporation and cultivation of THP-1 cells in HA–TA : GTN–HPA hydrogels enhances monocyte-to-macrophage differentiation in these cells. This phenomenon is the most pronounced after 7 days of cultivation (Fig. 3).

### 3.4. Influence of HA content in the material on encapsulated cells

Besides adjusting the concentrations of the crosslinking agents, the system can be also modified by changing the HA–TA : GTN–HPA ratio. The influence of different ratios of the components used was tested on hydrogels prepared with the following concentration of the agents: 0.56 U ml^−1^ of HRP and 2.31 mM of H_2_O_2_. The following ratios of HA–TA : GTN–HPA were tested: 1 : 5, 1 : 10, and pure GTN–HPA.

**Fig. 4:**
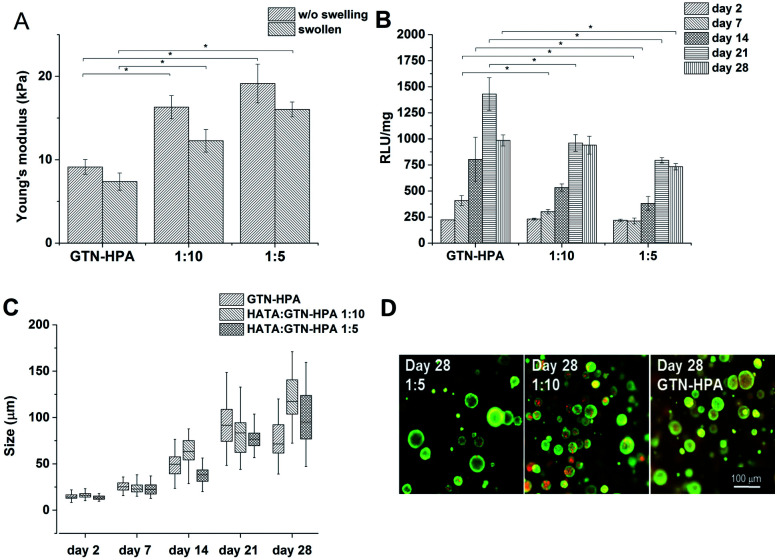
Influence of material composition on (A) Young's modulus (B) metabolic activity (C) average size of clusters formed by the THP-1 cells (D) morphology of the cells.

The gelation times for all the HA–TA : GTN–HPA ratios were less than 6 s; the *G*′ plateau was reached in less than 3 minutes in every case. With increasing content of GTN–HPA the Young‘s modulus decreased; degrees of syneresis were observed in the materials; the weight of the material with a ratio of 1 : 10 decreased by up to 30% and the weight of hydrogels produced from pure GTN–HPA decreased by up to 40% (Fig. 4A).

Cells were encapsulated in hydrogels with the 1 : 5 and 1 : 10 ratios, as well as in pure GTN–HPA. Cells in all the types of hydrogels formed clusters of similar sizes independently of the material composition, but metabolic activity increased with increasing GNT–HPA content (Fig. 4B–D).

**Fig. 5:**
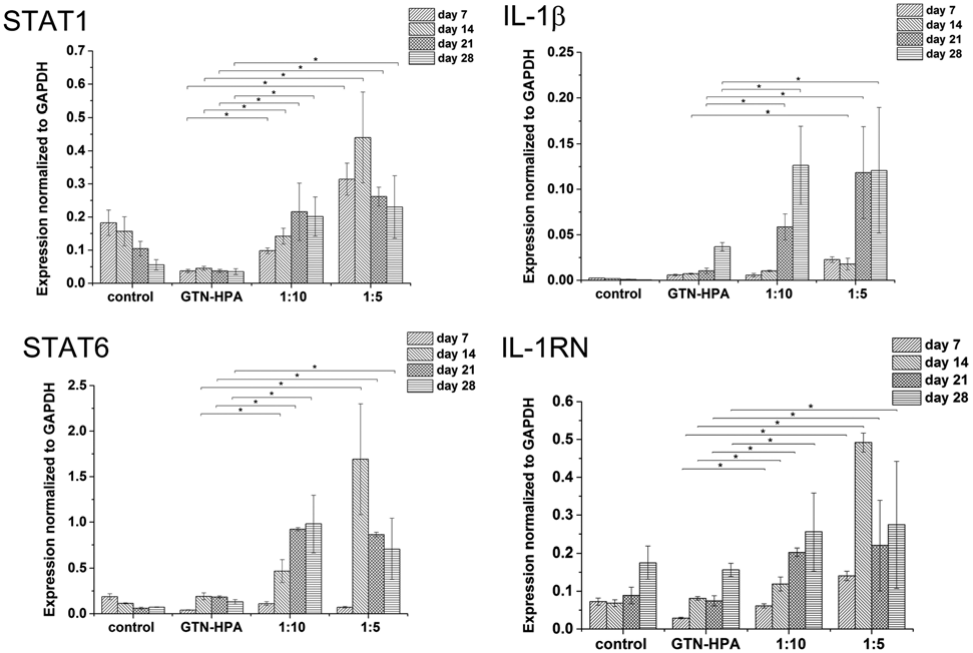
Expression of M1 marker genes STAT1, IL-1β, and M2 marker genes STAT6, IL-1RN was assessed in non-encapsulated cells (control) and in cells encapsulated in hydrogels prepared from pure GTN–HPA or mixtures of HA–TA : GTN–HPA.

Cells cultured in hydrogels with a content of HA–TA had overall higher expression of macrophage polarization related genes than cells cultured in pure GTN–HPA scaffolds or in non-encapsulated cells, while the total number of cells in hydrogels of different compositions was within the same range (Fig. 5). These data suggest that the presence of HA–TA in the material supported THP-1 differentiation into macrophages. The influence of HA–TA concentration on the macrophages polarization was not confirmed, because differences between the expression of M1 and M2 markers produced by cells encapsulated in hydrogels with different amounts of HA–TA (1 : 5 or 1 : 10) were not statistically significant.

**Fig. 6:**
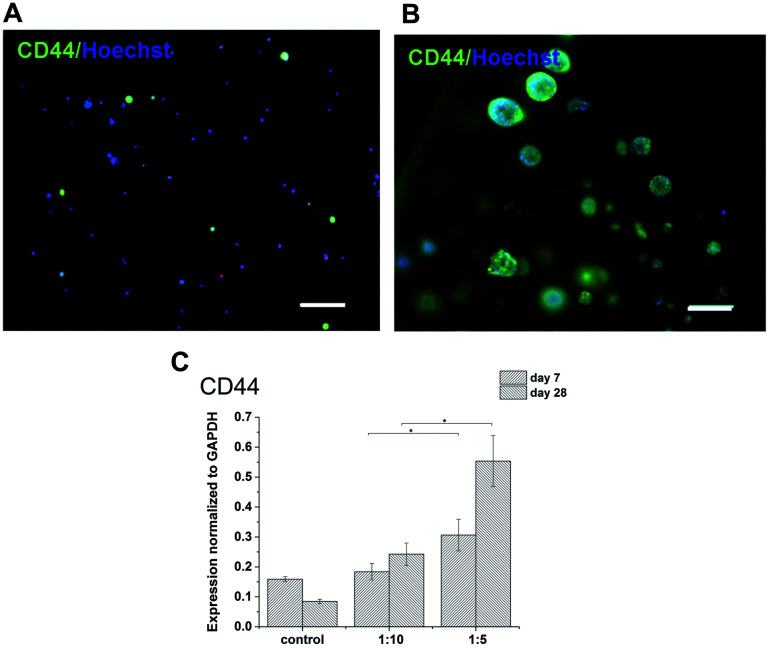
CD44 expression by THP-1 cells cultured (A) in a culture flask and (B) in hydrogel for 4 weeks. Immunofluorescent staining with anti-CD44 (green), counterstained with Hoechst (blue). Scale bar = 100 μm. (C) Expression of CD44 in the materials with different content of GTN.

The expression of HA receptor CD44 in cells cultured in the presence of HA (ratios of 1 : 5 and 1 : 10) was studied by real-time PCR and immunofluorescent staining (Fig. 6). For 28 days, the expression of CD44 increased slightly in the scaffolds with the lower amount of HA–TA (1 : 10) but increased significantly in the scaffolds with the higher amount of HA–TA (1 : 5). Immunofluorescent staining of the CD44 was positive in cells growing in scaffolds for 4 weeks and mostly negative for non-encapsulated cells (control).

### 3.5. Cell-mediated degradation of the material

In order to study cell-mediated degradation, the hydrogels with a high content of GTN–HPA (1 : 10) and a concentration of crosslinking agents of 2.31 mM of H_2_O_2_ and 0.56 U ml^−1^ of HRP were prepared. The hydrogel composition was chosen because it provides an environment supporting both proliferation and differentiation of encapsulated cells. Previous experiments proved that the material itself provides an environment that is able to partially induce cell differentiation into macrophages. In this experiment, macrophage differentiation was further supported by the differentiation agents PMA^24,25^ (an agent widely used for the differentiation of THP-1 cells into macrophages) and IL-4 (one of the main M2 polarization stimulating agents), PMA was used for 24 hours followed by 72 hours treatment by IL-4. The differentiation in the PMA/IL-4 treated cells manifested itself by an inhibition of proliferation, change in morphology (cells were enlarged, some were elongated or created pseudopodia), and enhancement of macrophage marker expression (Fig. 8). The expression of M1 markers and IL-1RN was triggered by PMA and later decreased, while the expression of M2 markers increased after IL-4 treatment. Non-differentiated monocytes proliferated rapidly and created clusters similar to those observed in the previous experiments.

**Fig. 7:**
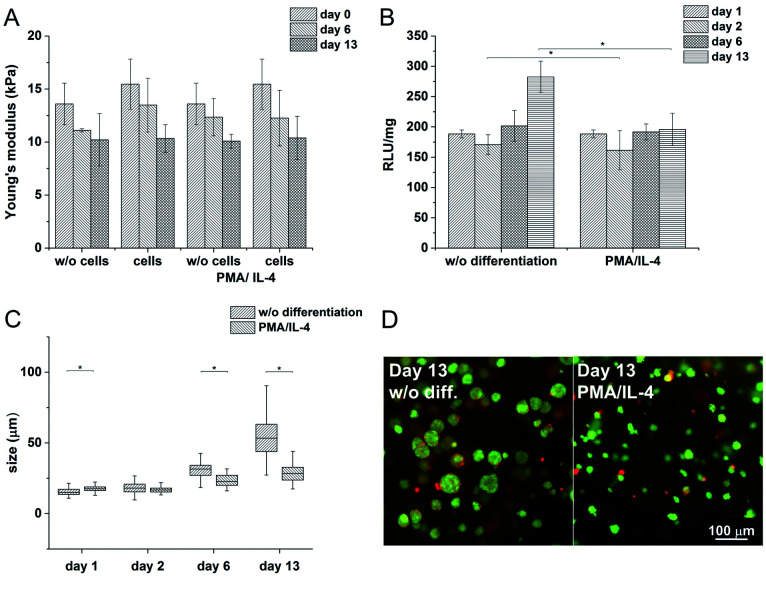
A) Influence of cells on the stiffness of the material during cultivation (B) metabolic activity of the cells without and with PMA/IL-4 induced differentiation (C) average sizes of clusters (D) differences in cell morphology after the induction of differentiation.

The Young's modulus decreased during the cultivation of the scaffolds (from *E* ≈ 15 kPa to *E* ≈ 10 kPa) independently of the presence of the cells (Fig. 7A). There was no apparent difference even after the induction of cell differentiation. It could be assumed that the metabolic activity of the cells did not affect the mechanical properties of the material within the given period of time.

**Fig. 8:**
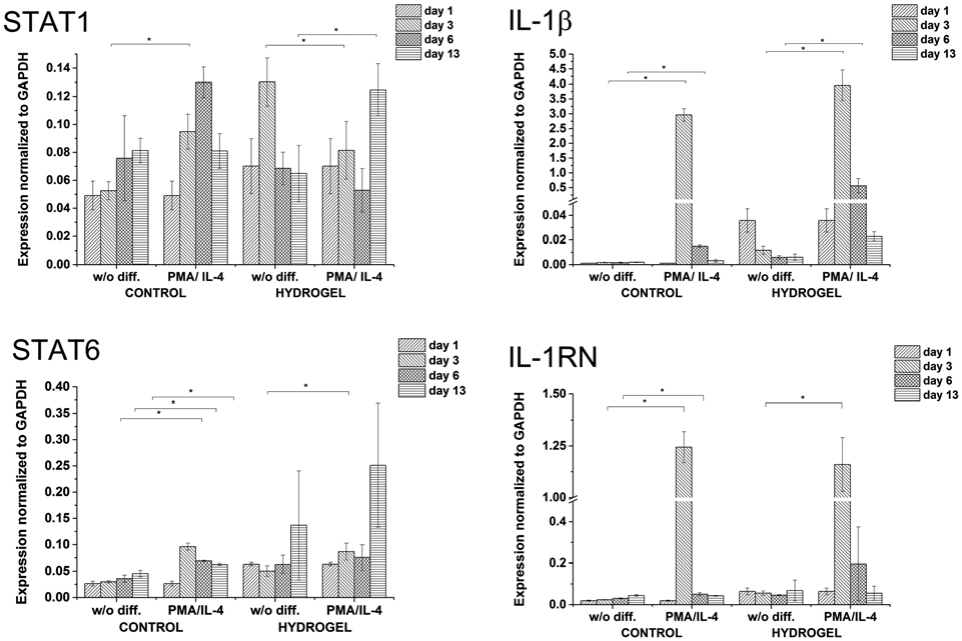
Expression of M1 marker genes STAT1, IL-1β, and M2 marker genes STAT6, IL-1RN in non-encapsulated (control) and encapsulated cells (hydrogel).

## 4. Discussion

The encapsulation of monocytes as a potential therapeutic agent has its foundation in tissue macrophages (or resident macrophages). Tissue macrophages are an integral component of many tissues and their activities are essential for the proper functioning and maintenance of tissues.^6,8^ Osteoclasts, Kupffer cells in the liver, microglia in the brain, alveolar macrophages, *etc.* play an active role in maintaining the integrity of tissues through their interactions with the other cells of their corresponding tissues.^29^ They are even more actively involved following an injury due to their ability to respond to an injury by reverting to a pro-inflammatory phenotype.^6,7,30^ During inflammation, resident macrophages are supplemented by incoming macrophages; however, in the case of an engineered tissue, the newly introduced artificial tissue has no resident macrophage component.^31^ As it was recently shown that resident macrophages are in most cases established before birth,^29^ it is important to (i) have the necessary means to recruit monocytes from blood *via* cytokines or chemoattractants or (ii) to supply the artificial tissue with monocytes during its *in vitro* maturation. Thus, the incorporation of monocytes and macrophages into artificial tissues is a logical next step similar to the addition of endothelial cells for the induction of angiogenesis. Moreover, as 3D microtissue models have become more common in cell culture studies, the microenvironment described here can also be used for the addition of an immune cell component to artificial tissues to render them “immunocompetent”.

As soft tissues have a wide range of mechanical properties, it is advantageous to develop structures based on ECM components that can exhibit a similar range of degrees of stiffness and related properties. In this work, systems based on hydrogels composed of derivatives of hyaluronic acid and gelatine were designed and optimized to provide a microenvironment for monocyte encapsulation and differentiation.

Hydroxyphenyl derivatives of HA and GTN were chosen as raw materials for hydrogel preparation in this study. These derivatives can undergo horseradish peroxidase mediated crosslinked reaction. This process was successfully used for encapsulation of living cells in hydrogel matrix, recently.^32–34^

One of the main advantages of using these derivatives is their short gelation time, in the order of seconds, which makes them efficient as injectable cell delivery medium. Hydrogels containing different concentration ratio of HA–TA and GTN–HPA component were tested in order to describe the influence of material composition on the stiffness and swelling stability of the hydrogels, cell growth, proliferation, and differentiation. The obtained data showed that hydrogels based on modified GTN and HA–TA have a significantly higher stiffness (*E* ≈ 19 kPa) and better volume stability than hydrogels based only on GTN–HPA (*E* ≈ 10 kPa). The values of the Young's modulus and volume stability are dependent on the content of gelatine in the HA–TA : GTN–HPA mixture: the swelling ratio and volume stability decreased with decreasing amount of the HA derivative. Thus, the addition of co-crosslinkable HA does not only produce a more faithful microenvironment, but it also provides improved stability which is important for applications involving encapsulated cell delivery.

The effect of hydrogel stiffness on cell behaviour is well documented,^28,35,36^ although to our best knowledge, the effect of 3D encapsulation of monocytes in structures of different degrees of stiffness has not been dealt with in published literature yet. It has been previously shown that THP-1 cells can be used as a representative model of human macrophages.^37^

We demonstrated that encapsulation of naïve monocytes does not affect their viability and that being encapsulated had a direct effect on their phenotype.

Our study revealed that hydrogel stiffness has a significant influence on monocyte growth and proliferation. Cells cultured in soft hydrogels mimicked suspension-like growth and had high metabolic activity, while cells cultured in stiffer gels created dense clusters and had lower metabolic activity. Such a large-scale effect on monocyte morphology by the encapsulation milieu indicates that the utilization of ECM-mimicking environments can provide a means of monocyte/macrophage phenotype control with less reliance on cytokines.

Different material compositions (different HA–TA : GTN–HPA ratio) influenced cell proliferation and phenotype. Cell proliferation increased with increasing amount of GTN–HPA. Expression of CD44, the main surface molecule interacting with HA, in cells cultured in HA–TA : GTN–HPA hydrogels was higher compared to non-encapsulated cells. The presence of HA–TA in scaffolds induced the expression of CD44 in THP-1 cells and the induction was related to the amount of HA–TA in the hydrogel. This study further revealed that the encapsulation of monocytes in hydrogels containing HA–TA partially induced monocyte-to-macrophage differentiation. We observed slight differences in the expression of M1 and M2 marker genes among cells cultured in hydrogels composed of different HA–TA : GTN–HPA ratios. However, further experimental work and analysis are needed to see whether there an HA-concentration-dependent polarization occurs.

Besides material stiffness, volume stability and gelation time, degradation is another important factor which should be well defined and considered during the development of materials for tissue engineering. Some studies have shown that highly metabolically active monocytes can actively degrade hydrogels.^38^ This phenomenon was not observed in our system. Material properties, which were evaluated based on the Young's modulus, showed an identical trend under all tested conditions independently of cell presence in the hydrogel structure and even with induced differentiation. The decreasing stiffness of the material corresponded with changes in the structure of the material during cultivation. Due to this fact, it is possible to assume that the material, if implanted *in vivo*, would degrade within weeks which would allow full tissue regeneration.

## 5. Conclusions

We developed and optimized a hydrogel system suitable for 3D cultivation of monocytes that fully supports their proliferation and viability. It can be used as a microenvironment for *in vitro* studies of immune cells, as a potential immunomodulatory agent for tissue engineering applications or as a coating for medical devices. Moreover, its adjustable properties and ECM-mimicking composition make our material suitable for enhancing the biocompatibility of various implants without interfering with their integration.

## Conflicts of interest

Vladimír Velebný has a financial interest in the company Contipro, Dolní Dobrouč, Czech Republic, the commercial producer of hylauronan. Julie Bystroňová, Ivana Ščigalková and Martin Pravdaare employees of Contipro.

## Supplementary Material

RA-008-C7RA13739G-s001
