# Supplementary material for: Creating a 3D microenvironment for monocyte cultivation: ECM-mimicking hydrogels based on gelatine and hyaluronic acid derivatives
Source: RSC Adv. 2018 Feb 16;8(14):7606–14. doi: 10.1039/c7ra13739g (PMC9078457; doi:10.1039/c7ra13739g)
Supplement: RA-008-C7RA13739G-s001 [file RA-008-C7RA13739G-s001.pdf]

Electronic Supplementary Material (ESI) for RSC Advances.
This journal is © The Royal Society of Chemistry 2018



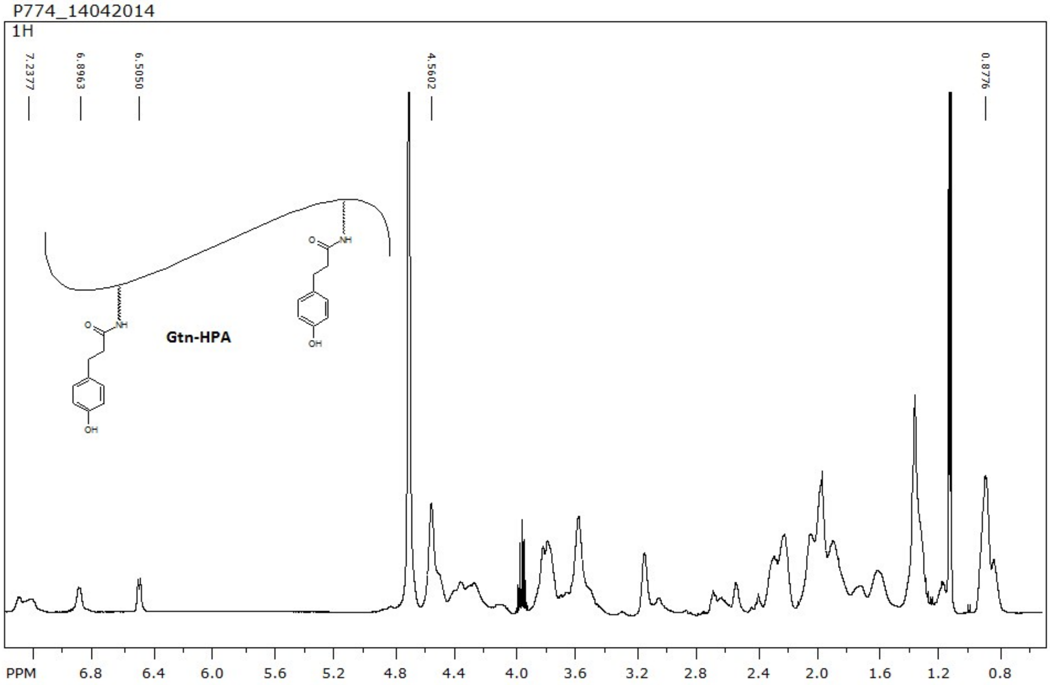


**Figure S1: $^1$H NMR spectrum of Gtn-HPA Signals assignment -** $^1$H NMR: ($D_20$, ppm) δ:0.88 – 4.56 (Gtn backbone); 6.50 (d, 2 H arom of HPA); 6.90 (s, 2 H arom of HPA); 7.15 – 7.35 (aromatic acid residues of Gtn backbone).

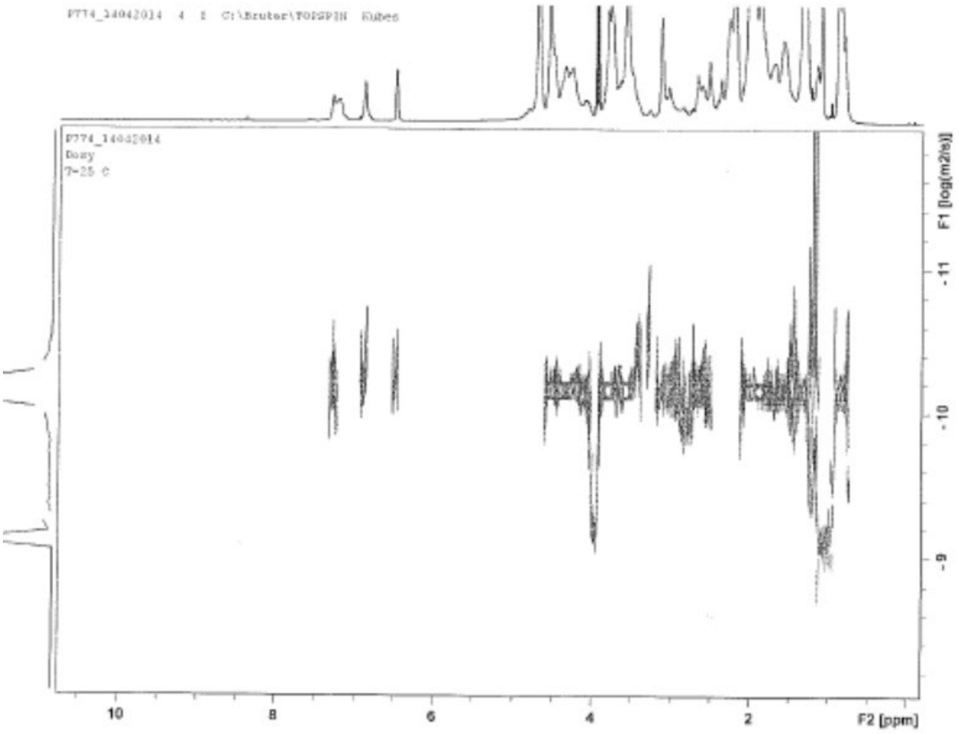


**Figure S2: DOSY spectrum of Gtn-HPA**

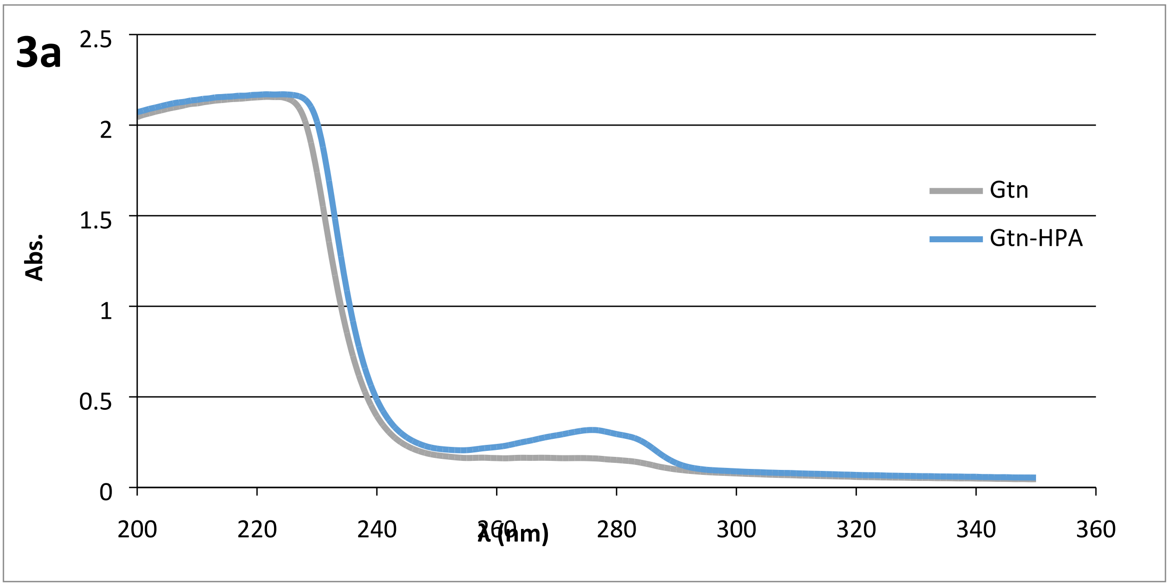


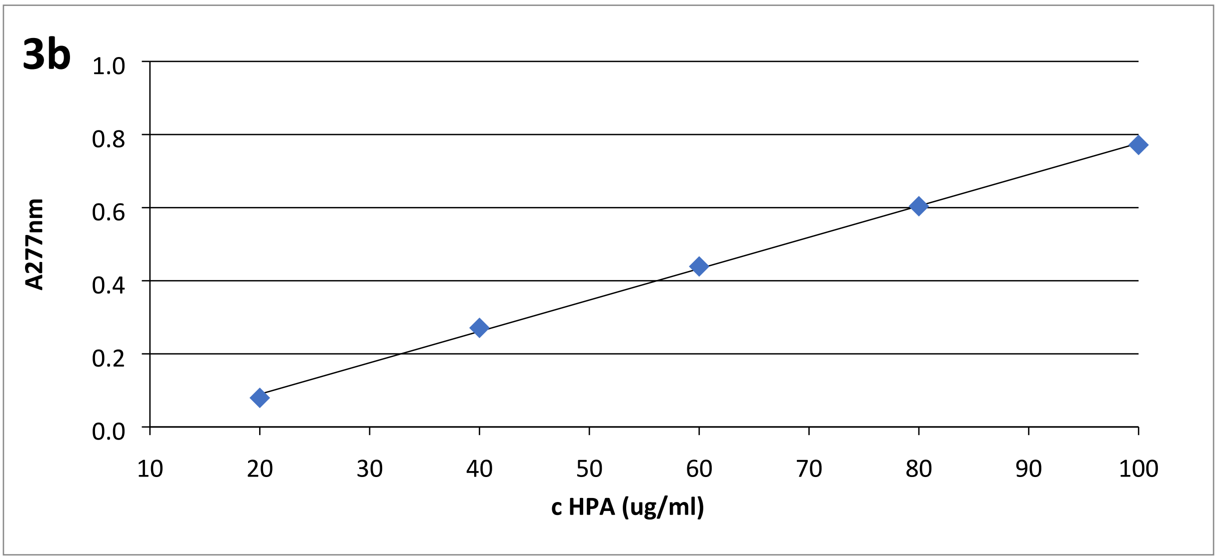


**Figure S3: a) UV/VIS spectra of prepared Gtn-HPA conjugate b) calibration curve of HPA in the solution of gelatin (2 mg/ml)**

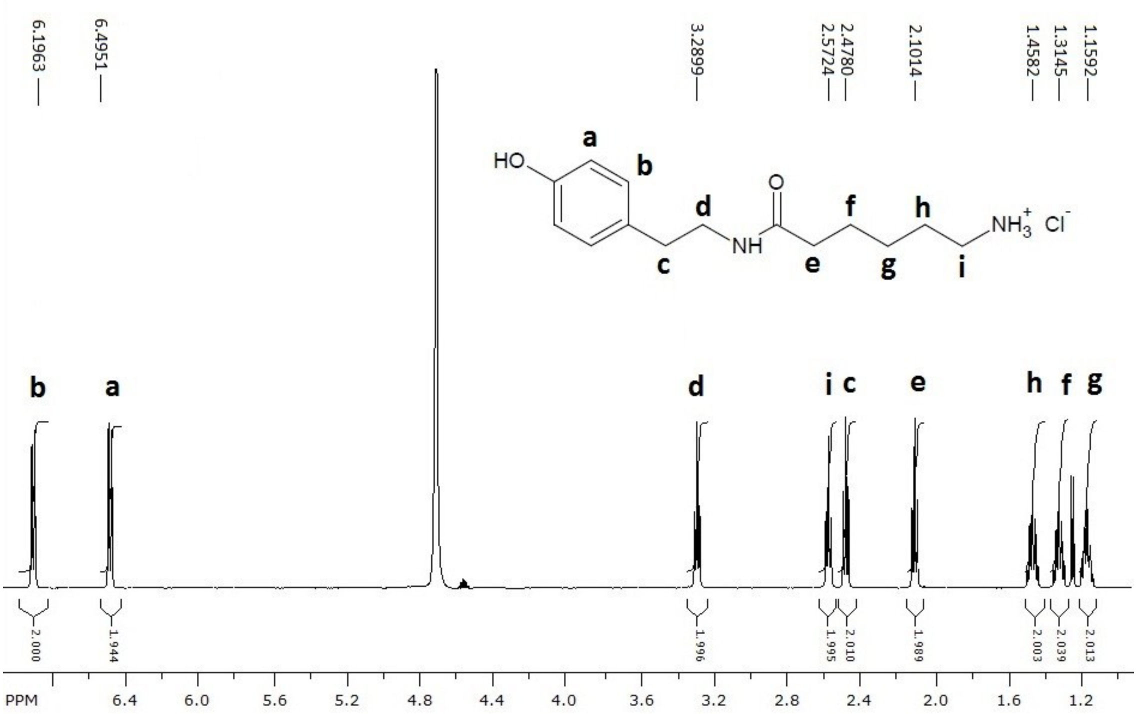


**Figure S4: $^1$H NMR spectrum of 6-amino-N-[2-(4-hydroxyphenyl)ethyl]hexanamide (Ahx-TA). Signals assignment:**$^1$H NMR: ($D_2$0+NaOD, ppm) δ: 1.16 (m, 2 H, γ-$CH_2$- hexanoic acid); 1.31 (m, 2 H, β-$CH_2$-hexanoic acid); 1.46 (m, 2 H, δ-$CH_2$-hexanoic acid); 2.10 (t, 2 H, -$CH_2$-CO-); 2.48 (t, 2 H, -$CH_2$-Ar); 2.78 (t, 2 H, -$CH_2$-$NH_2$); 3.29 (t, 2 H, -$CH_2$-NH-CO); 6.50 (d, 2 H, C6-arom); 6.90 (s, 2 H, C4-arom).

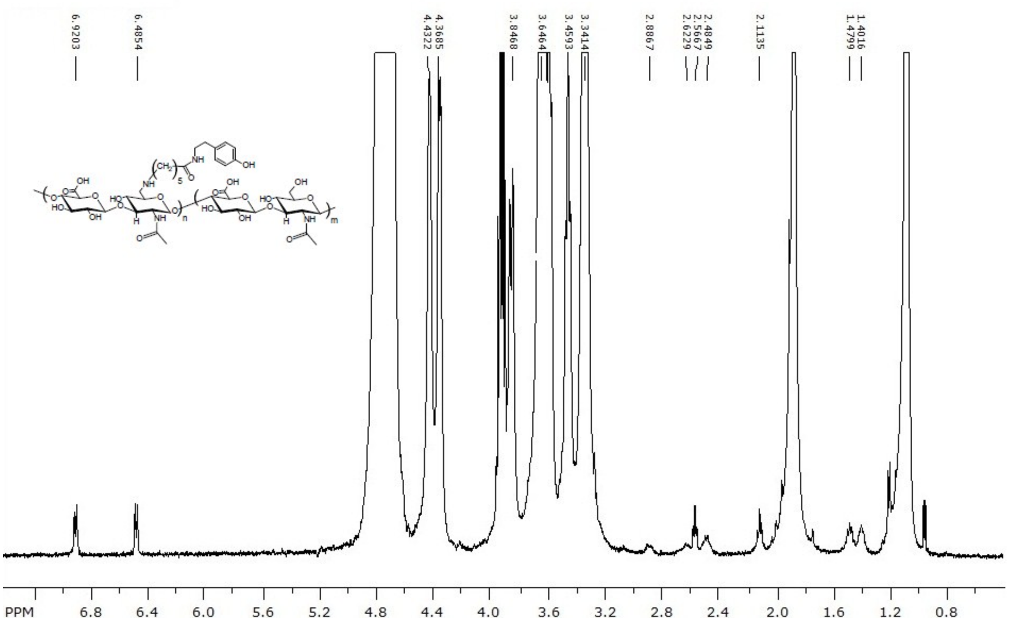


**Figure S5: $^1$H NMR spectrum of HA-TA Signals assignment:**HA- TA $^1$H NMR (D20+NaOD) δ: 1.20 (m, 4 H, γ-CH2-hexanoic acid); 1.33 - 1.40 (m, 4 H, β-CH2-hexanoic acid); 1.46 (m, 4 H, δ-CH2-hexanoic acid);, 2.01 (s, 3 H, CH3-CO), 2.11 (t, 2H, -CH2-CO-), 2.49 (m, 2 H, -CH2-NH-HA), 2.56 (t, 2 H, Ph-CH2-CH2-), 2.63 and 2.89 (diastereotopic 2H, HA-CH2-NH-), 3.27 (t, 2 H, Ph-CH2-CH2-), 3.37 – 3.86 (m, 10 H, hyaluronan body), 4.36 (s, 1H, anomer O-CH(OH)-), 4.43 (s, 1H anomer., O-CH(OH)-), 6.49 (d, 2H, arom.), 6.92 (d, 2H. arom.).
